# Genome-wide association analysis identifies a candidate gene controlling seed size and yield in *Xanthoceras sorbifolium* Bunge

**DOI:** 10.1093/hr/uhad243

**Published:** 2023-11-22

**Authors:** Ziquan Zhao, Chongjun Liang, Wei Zhang, Yingying Yang, Quanxin Bi, Haiyan Yu, Libing Wang

**Affiliations:** State Key Laboratory of Tree Genetics and Breeding, Research Institute of Forestry Chinese Academy of Forestry, Beijing 100091, China; State Key Laboratory of Tree Genetics and Breeding, Research Institute of Forestry Chinese Academy of Forestry, Beijing 100091, China; College of Forestry, Hainan University, Haikou 570228, China; State Key Laboratory of Tree Genetics and Breeding, Research Institute of Forestry Chinese Academy of Forestry, Beijing 100091, China; State Key Laboratory of Tree Genetics and Breeding, Research Institute of Forestry Chinese Academy of Forestry, Beijing 100091, China; State Key Laboratory of Tree Genetics and Breeding, Research Institute of Forestry Chinese Academy of Forestry, Beijing 100091, China; State Key Laboratory of Tree Genetics and Breeding, Research Institute of Forestry Chinese Academy of Forestry, Beijing 100091, China; State Key Laboratory of Tree Genetics and Breeding, Research Institute of Forestry Chinese Academy of Forestry, Beijing 100091, China; College of Forestry, Hainan University, Haikou 570228, China; College of Forestry, Northwest A&F University, Yangling 712100, China

## Abstract

Yellow horn (*Xanthoceras sorbifolium* Bunge) is a woody oilseed tree species whose seed oil is rich in unsaturated fatty acids and rare neuronic acids, and can be used as a high-grade edible oil or as a feedstock for biodiesel production. However, the genetic mechanisms related to seed yield in yellow horn are not well elucidated. This study identified 2 164 863 SNP loci based on 222 genome-wide resequencing data of yellow horn germplasm. We conducted genome-wide association study (GWAS) analysis on three core traits (hundred-grain weight, single-fruit seed mass, and single-fruit seed number) that influence seed yield for the years 2022 and 2020, and identified 399 significant SNP loci. Among these loci, the Chr10_24013014 and Chr10_24012613 loci caught our attention due to their consistent associations across multiple analyses. Through Sanger sequencing, we validated the genotypes of these two loci across 16 germplasms, confirming their consistency with the GWAS analysis results. Downstream of these two significant loci, we identified a candidate gene encoding an AP2 transcription factor protein, which we named *XsAP2*. RT–qPCR analysis revealed high expression of the *XsAP2* gene in seeds, and a significant negative correlation between its expression levels and seed hundred-grain weight, as well as single-fruit seed mass, suggesting its potential role in the normal seed development process. Transgenic *Arabidopsis* lines with the overexpressed *XsAP2* gene exhibited varying degrees of reduction in seed size, number of seeds per silique, and number of siliques per plant compared with wild-type *Arabidopsis*. Combining these results, we hypothesize that the *XsAP2* gene may have a negative regulatory effect on seed yield of yellow horn. These results provide a reference for the molecular breeding of high-yielding yellow horn.

## Introduction

Seeds serve as the foundation for plant reproduction and the continuity of generations. Larger seeds are thought to enhance the ability of plant seedlings to withstand external stresses and promote seedling survival, while plants with smaller seeds compensate for the relatively lower individual seed survival rate by producing a larger quantity of seeds [1]. Seed size plays a critical role in plant adaptation to the environment and significantly influences crop yield [2]. The regulation of seed size genes and genetic networks has been comprehensively studied. Plants regulate seed size through two primary mechanisms: maternal tissue-mediated control and syncytial tissue-mediated control. Several signaling pathways have been identified as regulators of seed size via modulation of maternal tissue growth. These pathways encompass the MAPK signaling pathway, the G-protein signaling pathway, the ubiquitin-proteasome pathway, phytohormone induction, and transcription factor regulation. The MAPK cascade reaction involves three classes of protein kinases: an MAPK kinase kinase (MKKK), an MAPK kinase (MKK), and an MAPK [3]. *Arabidopsis thaliana MKK4* and *MKK5* govern seed embryo development, and the double mutants display the characteristic of reduced seed size [4]. The heterotrimeric G protein complex comprises Gα, Gβ, and Gγ subunits. Overexpression of the *A. thaliana AGG3* gene, which encodes the Gγ subunit, leads to an increase in seed and organ size, while *agg3* mutants exhibit markedly reduced seed and organ sizes, indicating the promotion of *Arabidopsis* seed and organ growth by *AGG* [5, 6]. *DA1*, an *Arabidopsis* ubiquitin receptor, exerts negative regulation on seed size by modulating cell proliferation in the seed coat [7], while *DA2* and *ENHANCER OF DA1* (*EOD1*)/*BIG BROTHER* (*BB*) negatively regulate seed size through their interaction with *DA1* [8, 9]. It has been demonstrated that brassinosteroids (BRs) and auxins (IAA) play a role in regulating seed growth. The *Arabidopsis BSU1* gene is involved in BR signaling and promotes cell elongation and division [10]. Within the transcription factor regulatory pathway, the *OsGRF4* gene primarily enhances seed size by promoting cell expansion and, to a lesser extent, cell proliferation [11]. *APETALA2* (*AP2*), through the maternal sporophyte and endosperm genome, controls seed weight and seed yield [12, 13]. Syncytial tissue growth additionally influences seed size, with studies indicating the involvement of the *HAIKU* (*IKU*) pathway and certain phytohormones in the regulation of seed size through their impact on endosperm development. *Arabidopsis haiku1* (*iku1*), *iku2*, and *miniseed3* (*mini3*) mutants produce small seeds. Pollination of these mutants with wild-type (WT) pollens results in normal-sized seeds, suggesting that *IKU1*, *IKU2*, and *MINI3* function zygotically to regulate seed growth [14, 15].

Yellow horn (*Xanthoceras sorbifolium* Bunge) is a woody oilseed tree species, with its seeds being the primary oil-producing organs. The seed oil is characterized by exceptionally high levels of unsaturated fatty acids, especially oleic acid (~40%) and linoleic acid (~30%). Of greater significance, yellow horn oil contains ~3% of nervonic acid, a unique component not found in other plant oils [16]. Nervonic acid plays a crucial role in repairing damaged neural cells and promoting infant brain development [17, 18]. In addition to its application as an edible oil, yellow horn oil serves as an excellent raw material for biodiesel production [16, 19, 20]. In addition, the oil-extracted seed cake, due to its abundant protein content, can also serve as feed for livestock or pets [19]. Therefore, yellow horn seeds have great commercial value [17].

Regrettably, compared with other major woody oilseed tree species, such as *Idesia polycarpa* Maxim., *Juglans regia* L., and *Canarium oleosum*, the yield of yellow horn remains relatively low, which constitutes one of the limiting factors for its development. Therefore, enhancing seed production is a crucial and urgent breeding objective for yellow horn. Substantial efforts have been made to improve yellow horn seed yield in recent times. For instance, at the physiological level, one study has shown that the quality of male parent pollen influences fruit set rate, and artificial selection through pollination can increase fruit set, consequently boosting seed production [21]. Another investigation has revealed a highly significant negative correlation between seed size and altitude, suggesting the potential for increasing seed yield through geographic transplantation [22]. A separate study also explored the impact of the canopy microclimate on seed yield, observing a significant positive correlation between light intensity, temperature, and seed yield [23]. Moreover, at the molecular genetic level, the ethylene receptor gene (*XsERS*) and the superoxide dismutase gene (*XsSOD*) have been identified as candidate genes affecting early ovule development [24, 25]. Certain miRNA interaction modules, such as *miR172b-ARF2* and *miR7760-p3_1-AGL61*, have been identified as potential regulatory modules for seed development and lipid synthesis [26]. In summary, research on yellow horn seed yield is currently limited, with most studies focusing on physiological aspects. Even in the realm of molecular genetics, research has primarily stopped at candidate gene identification, lacking further functional validation and exploration of regulatory mechanisms.

However, seed yield is a complex trait influenced by multiple factors, making it challenging to comprehensively address all aspects. In this study, we focused specifically on three crucial factors, hundred-grain weight (HWG), seed mass of single fruit (SFSM), and seed number of single fruit (SFSN), which significantly influence seed yield. Based on resequencing data from 222 yellow horn germplasms, we first explored the population structure and kinship relationships among these germplasms, and then performed genome-wide association analysis on the phenotypic data of these three traits over two years (2022 and 2020). By analyzing significant SNP loci, we successfully identified a candidate gene that regulates both hundred-grain weight and single-fruit seed mass. Subsequently, we verified the function of this gene in detail through *Arabidopsis* transgenic, RT–qPCR, subcellular localization, and seed embryo microscopic observation experiments. These findings provide important insights for investigating seed size in yellow horn and for molecular breeding of high-yielding cultivars.

## Results

### Phenotypic variation and correlation analysis for agronomic traits

**Figure 1:**
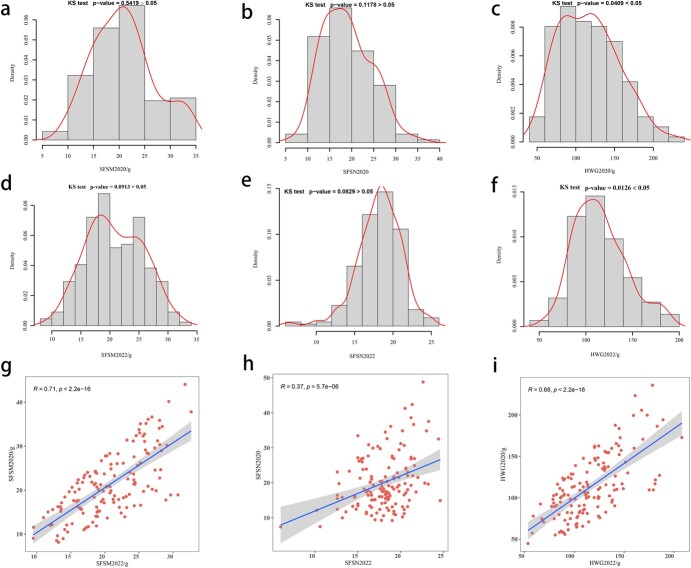
**a**–**f** Density distribution plots of HWG, SFSN, and SFSM phenotypic data for 2020 and 2022, with curved lines indicating the corresponding normal distribution curves. **g–i** Scatterplots of the data for the three phenotypes in 2020 and 2022, with the straight line indicating the linear fit curve and the shaded area indicating the 95% confidence interval.

We assessed normality for each of the three phenotypes in both years. Based on Kolmogorov–Smirnov test results, only HWG in 2020 (HWG2020; *P* = .04) and HWG2022 (*P* = .01) phenotypes demonstrated normal distribution (Fig. 1c and f). However, SFSN in 2020 (SFSN2020; *P* = .11), SFSN2022 (*P* = .08), and SFSM in 2020 (SFSM2022; *P* = .09) did not conform to a normal distribution but exhibited characteristics of a skewed normal distribution (Fig. 1b, e, and d). Conversely, the distribution of SFSM2020 (*P* = .54) phenotypic data exhibited a notable departure from normality (Fig. 1a). Furthermore, linear regression analyses conducted over various years for the three phenotypes revealed more robust regression results for the SFSM (*R* = 0.71) and HWG (*R* = 0.68) phenotypes, implying a high degree of genetic stability in these traits. Conversely, the SFSN (*R* = 0.37) phenotype exhibited weaker regression, possibly due to its susceptibility to environmental influences (Fig. 1g–i).

### Resequencing of yellow horn germplasm resources and variant discovery

The genomic DNA from 222 yellow horns was resequenced, generating a total of 1690.7 Gb of raw data, with an average of 7.6 Gb per sample, 1675.5 Gb of filtered cleaned data, an average of 7.547 Gb of cleaned data per sample, an average Q30 ratio of 93.8%, and an average GC content of 39.3% (Supplementary Data Table S1).

**Figure 2:**
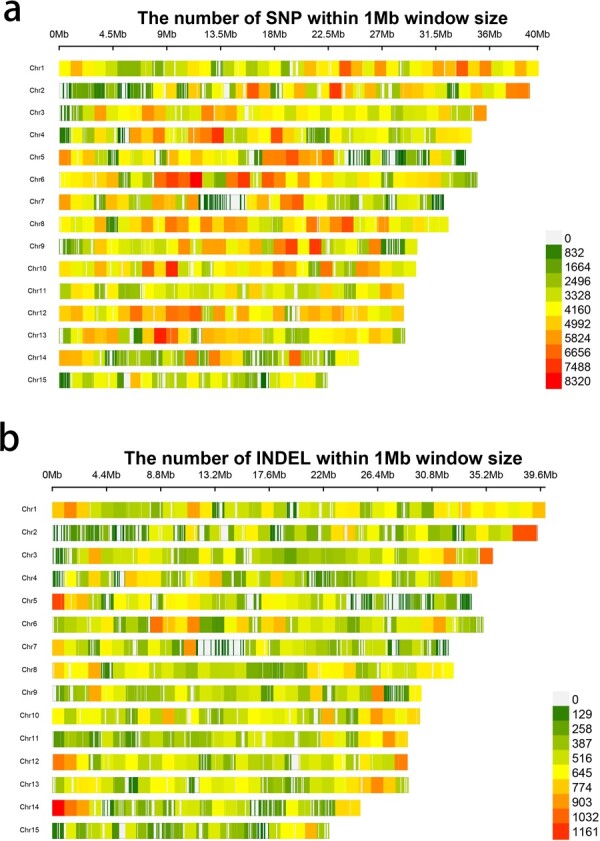
**a** Distribution of SNP sites on 15 chromosomes. **b** Distribution of indel sites on 15 chromosomes.

The ZS4 genome was employed as the reference to align the resequenced data from 222 samples, facilitating the identification of genome-wide variant sites. Following rigorous quality control and screening, we obtained a total of 2 164 863 high-quality variant sites, comprising 1 926 312 SNP sites and 238 551 indel sites (Fig. 2a and b). The distribution of these variants across the genome exhibited heterogeneity, with the majority residing in non-coding regions (983 930, 45.45%). Additionally, 168 210 (7.77%) of the variant loci within coding regions were situated in exon regions, and 260 650 (12.04%) were in intron regions. Notably, ~97 394 (57.9%) of the SNPs within exon variant loci led to non-synonymous mutations (Supplementary Data Table S2). These high-quality loci hold substantial significance for investigating the genetic underpinnings of yellow horn traits.

### Principal component analysis and linkage disequilibrium decay distance

**Figure 3:**
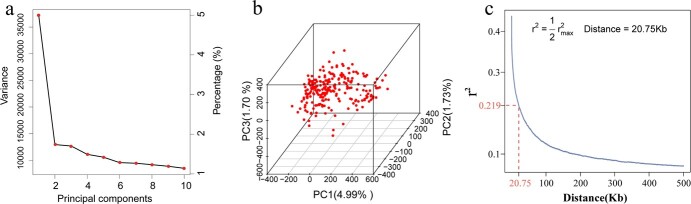
**a** Scatter plot depicting the variance explained by the first 10 principal components. **b** 3D PCA plot of the first three principal components. Each dot represents a sample. **c** LD decay plot. The LD decay distance is defined as the distance at which the LD coefficient *r*^2^ reaches half of its maximum value. The LD decay distance for the yellow horn is 20.75 kb, denoted by a dashed line.

To further investigate the genetic basis of these three agronomic traits, we initially conducted principal component analysis (PCA) on the genotype dataset to explore potential population stratification effects. The results indicated that the Bayesian information criterion (BIC) achieved the highest value when three principal components were included. This suggests that, for subsequent genome-wide association study (GWAS) analysis, correcting for population stratification by employing the first three principal components is the most suitable approach to mitigating the impact on the results. The first three principal components of the PCA explained 4.99, 1.73, and 1.70% of the phenotypic variation, respectively. Furthermore, the subsequent fourth to tenth principal components accounted for <1.5% of the total variance (Fig. 3a). To visually illustrate the influence of the first three principal components on the population, a 3D plot of the PCA was generated (Fig. 3b). The results indicated that all the sample points clustered together without distinct separation, suggesting the absence of significant population stratification among the studied samples. This finding aligns with the fact that all samples originate from a common germplasm resource base.

To determine the screening range of candidate genes, linkage disequilibrium (LD) analysis was performed using the genotype dataset (Fig. 3c). The LD decay distance was set to half of the maximum LD distance. The results revealed an LD distance of 20.75 kb for yellow horn.

### Genome-wide association studies for three agronomic traits

**Figure 4:**
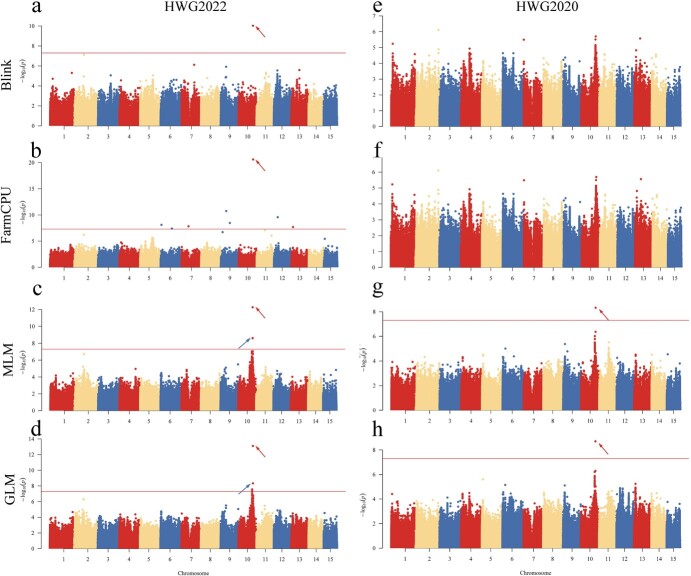
Manhattan plots of association results of BLINK, FarmCPU, MLM, and GLM models for HWG2020 and HWG2022 traits. The horizontal straight line indicates the threshold of significant association (7.64). The locus indicated by the arrow pointing to the upper left is Chr10_24013014. The locus indicated by the arrow pointing to the upper right is Chr10_24012613.

**Figure 5:**
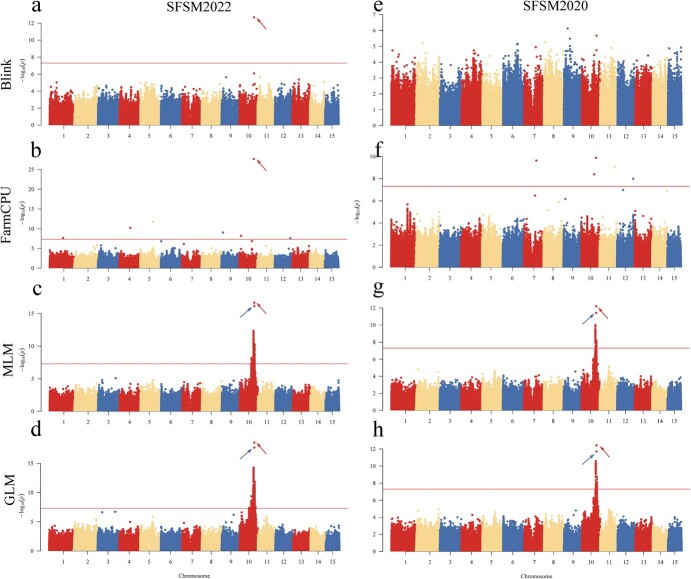
Manhattan plots of association results of BLINK, FarmCPU, MLM, and GLM models for SFSM2020 and SFSM2022 traits. The horizontal straight line indicates the threshold of significant association (7.64). The locus indicated by the arrow pointing to the upper left is Chr10_24013014. The locus indicated by the arrow pointing to the upper right is Chr10_24012613.

GWAS analysis was conducted for each agronomic trait using Bayesian-information and Linkage-disequilibrium Iteratively Nested Keyway (BLINK), Fixed and random model Circulating Probability Unification (FarmCPU), Mixed Linear Model (MLM), and General Linear Model (GLM). To control for potential false positives arising from group stratification effects, the first three principal components and the kinship matrix were included as covariates (Fig. 3b, Supplementary Data Fig. S1). The results of the GWAS analysis are presented in Manhattan plots (Figs 4 and 5, Supplementary Data Fig. S2) and QQ plots (Supplementary Data Fig. S3). A significance threshold of 7.64 [−log10 (0.05/2164863)] was applied to identify significant SNP loci associated with the traits. In the case of three traits, the HWG and SFSM traits exhibited associations with a considerable number of significant SNP loci, whereas the SFSN trait did not exhibit associations with any significant loci. Notably, the significant loci associated with the HWG and SFSM traits were located in the same chromosomal region, and a substantial proportion of these loci were redundant. In the case of the two years, the association results for the three traits in 2022 surpassed those from 2020. HWG2022 exhibited associations with 6 more loci than HWG2020, and SFSM2022 exhibited associations with 173 more loci than SFSM2020. Regrettably, both SFSN2022 and SFSN2020 did not exhibit associations with any significant loci, possibly due to the limited genetic influence on seed quantity in yellow horn single fruit. Among the four association models, MLM and GLM produced similar association results, as did the BLINK and FarmCPU models. However, the MLM and GLM models yielded a significantly higher number of associated loci than the BLINK and FarmCPU models. Interestingly, while the BLINK and FarmCPU models had fewer associated loci, they produced more statistically significant results for core loci. As the SFSN trait did not exhibit associations with significant loci and a substantial overlap was observed in the associated loci between the SFSM and HWG traits, we merged all association results and removed redundancy, resulting in 399 significant SNP loci (Supplementary Data Table S4).

Leveraging the genomic annotation of yellow horn and LD decay distance, we probed for candidate genes within a 20.75-kb range upstream and downstream of the significant SNP loci, resulting in the discovery of 72 candidate genes. Notably, the relative positional distribution of the significant SNP loci and candidate genes demonstrated marked imbalance: 68% of the significant SNP loci were located in the upstream or downstream regions of genes, 10% within gene exons, and 10% within introns, and 12% of the significant SNP loci had no corresponding identified candidate gene. Subsequently, we conducted a BLAST analysis using the NCBI online server for the protein sequences of all candidate genes, revealing their predominant encoding of protein kinases, calcium-binding proteins, hormone response factors, and transcription factors (Supplementary Data Table S4). These findings suggest the critical involvement of regulatory factors in determining seed size and single fruit yield in yellow horn.

### Identification of candidate genes involved in hundred-grain weight and single-fruit seed mass

**Figure 6:**
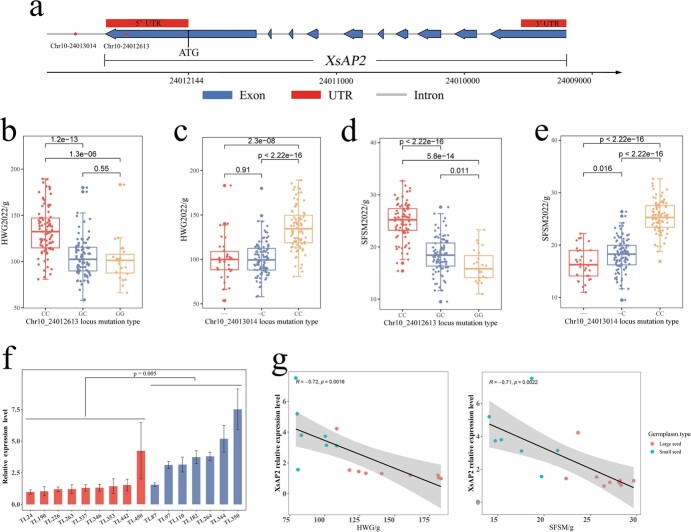
**a** Structural diagram of the *XsAP2* gene. Blue bars represent exons, red bars represent UTR regions, gray lines represent introns, and lines with arrows indicate chromosomal localization. **b–e** Box plots of phenotypic data for different genotypes at Chr10_24013014 and Chr10_24012613 loci. The central line of each boxplot represents the median of the data, while the upper and lower boundaries of the box represent the upper and lower quartiles of the data. **f** Relative expression levels of the *XsAP2* gene during the seed (DAP40) stage. Red columns represent large seed germplasms, while blue columns represent small seed germplasms. **g** Scatter plot of relative expression of the *XsAP2* gene and phenotype (HWG and SFSM), with the straight line indicating the linear fit curve and the shaded area indicating the 95% confidence interval.

Among the numerous significant SNP loci, the Chr10_24013104 and Chr10_24012613 loci have garnered substantial interest due to their consistent association across multiple GWAS analyses. Our investigation involved 16 GWAS analyses targeting two agronomic traits, namely, hundred-grain weight (HWG2022 and HWG2020) and single-fruit seed mass (SFSM2022 and SFSM2020), utilizing four distinct association models (BLINK, FarmCPU, MLM, and GLM). In 16 GWAS analyses, the Chr10_24013014 locus was identified as a significant site in 12 instances, with *P*-values ranging from 2.01E−09 to 2.12E−28. Similarly, the Chr10_24012613 locus exhibited significance in six GWAS analyses, with *P*-values ranging from 2.50E−09 to 1.99E−18 (Supplementary Data Table S4). Notably, all *P*-values for these two loci were well below the threshold for significant association (2.31E−08). These results substantiate the considerable impact of these loci on HWG and SFSM traits in the yellow horn. Further scrutiny unveiled that the Chr10_24013014 locus resided 870 bp upstream of the *EVM0013598* gene, whereas the Chr10_240126131 locus was situated within the 5′ untranslated region of the same gene, 469 bp upstream from the gene start (Fig. 6a). Examining the phenotype data corresponding to different genotypes at these loci, we found that samples with the CC genotype at the Chr10_24013014 locus exhibited significantly higher seed weight and single-fruit seed quality compared with those with −C and −− genotypes (Fig. 6c and e). Similarly, samples with the CC genotype at the Chr10_24012613 locus displayed significantly higher values than those with GC and GG genotypes (Fig. 6b and d).

To explore the connection between the genotypes of the Chr10_24013014 and Chr10_24012613 loci and yellow horn seed size as well as single-fruit yield, we cloned the promoter region of the *EVM0013598* gene (−370 to −952 bp) from nine large germplasms and seven small seed germplasms. The outcomes revealed that the genotypes of the large seed germplasms at Chr10_24013014 and Chr10_24012613 were CC and CC, while the small seed germplasms at these loci were −C and GC (Supplementary Data Fig. S4). These findings align with the results of the GWAS analysis (Fig. 6b and c). Furthermore, we measured the relative expression levels of the *EVM0013598* gene during the seed (DAP40, 40 days after pollination) stage (Fig. 6f). The results indicated a significantly lower relative expression level of the *EVM0013598* gene in the large seed germplasms compared with the small seed germplasms (*P* = .005). Simultaneously, a correlation analysis was performed between the relative expression levels of the *EVM0013598* gene in these 16 germplasms and the phenotypic data (HWG and SFSM). The results demonstrated a strong negative correlation between the relative expression level of the *EVM0013598* gene and the HWG trait (*R* = −0.72) as well as the SFSM trait (*R* = −0.71). Combining these results, we infer that polymorphism in the Chr_24013014 and Chr10_24012613 loci affects the transcription activity of the *EVM0013598* gene, thereby influencing yellow horn seed size and single-fruit yield.

### *XsAP2* gene is a homologous gene of *APETALA2*

The protein sequence of the *EVM0013598* gene was subjected to a BLAST analysis, confirming its affiliation with the *AP2/ERF* transcription factor family. To further understand the function of the *EVM0013598* gene, a phylogenetic tree was constructed with this gene and the *AP2/ERF* gene family in *A. thaliana*. The results showed that this gene belongs to the *AP2* subfamily of the *AP2/ERF* transcription factor gene family. Further analysis found that this gene has the highest similarity (64%) with the *Arabidopsis APETALA2* (*AT4G36920*) gene (Supplementary Data Fig. S5). To facilitate subsequent research, we have aptly renamed this gene as *XsAP2*.

### Subcellular localization and tissue expression pattern of the *XsAP2* gene

**Figure 7:**
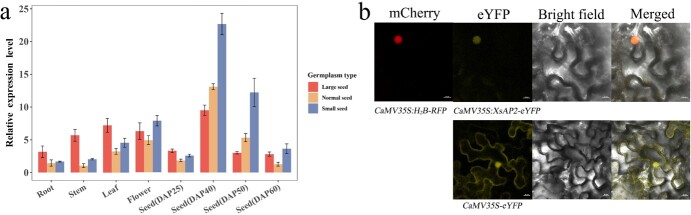
**a** Relative expression levels of the *XsAP2* gene in roots, stems, leaves, flowers, and seeds at different developmental stages of yellow horn. Red columns represent large-seed germplasm, yellow columns represent normal-seed germplasm, and blue columns represent small-seed germplasm. **b** Subcellular localization of the *XsAP2* gene in *N. benthamiana* leaf epidermal cells (scale bar, 10 μm).

The subcellular location of *XsAP2* was determined by fusing it with enhanced yellow fluorescent protein (eYFP) and inducing the expression of the fusion gene in *Nicotiana benthamiana* leaf epidermal cells, using the CaMV35S promoter. In the absence of the target gene sequence, eYFP fluorescent protein was expressed throughout *N. benthamiana* leaf epidermal cells. However, when the *XsAP2* gene was fused with eYFP, eYFP was expressed only in specific locations within the cell (Fig. 7b). In addition, we observed expression of the histone gene (*H2B*) fused with red fluorescent protein (RFP) at the same location (Fig. 7b); H2B-type histones are recognized as nucleus-localized proteins. The above results suggest that *XsAP2* is localized in the nucleus, consistent with the localization pattern of transcription factors.

The relative expression levels of the *XsAP2* gene in various tissues of yellow horn, including roots, stems, leaves, flowers, and seeds, were analyzed through RT–qPCR . Given the polymorphism of the Chr10_24013014 and Chr10_24012613 loci potentially affecting the expression of the *EVM0013598* gene, an analysis was conducted on small-seed germplasm, normal-seed germplasm, and large-seed germplasm. It is important to note that the genotypes for small-seed germplasm were −C_Chr10_24013014_ and GC_Chr24012613_ while the genotypes for large seed germplasm were CC_Chr10_24013014_ and CC_Chr24012613_, with other genotypes representing normal seed germplasm. The results revealed that the *XsAP2* gene exhibited extensive expression in various tissues and similar expression profiles among different types of germplasm (Fig. 7a). Interestingly, the *XsAP2* gene showed a pattern of increased and then decreased expression at different developmental stages of seed, with high expression levels at days 40 and 50 post-pollination. This pattern of expression mirrors the growth pattern of yellow horn seeds, which exhibit rapid growth from 25 to 50 days after pollination. Seed sizes practically stopped changing by day 60 following pollination, and the seeds transitioned from the growth stage to the maturity stage. The analysis of tissue expression profiles and subcellular localization led us to hypothesize that *XsAP2* gene expression is regulated at both spatial and temporal levels. Additionally, the gene could potentially affect the expression of specific genes, contributing to seed growth and development.

### Overexpression of the *XsAP2* gene reduces seed size and yield

**Figure 8:**
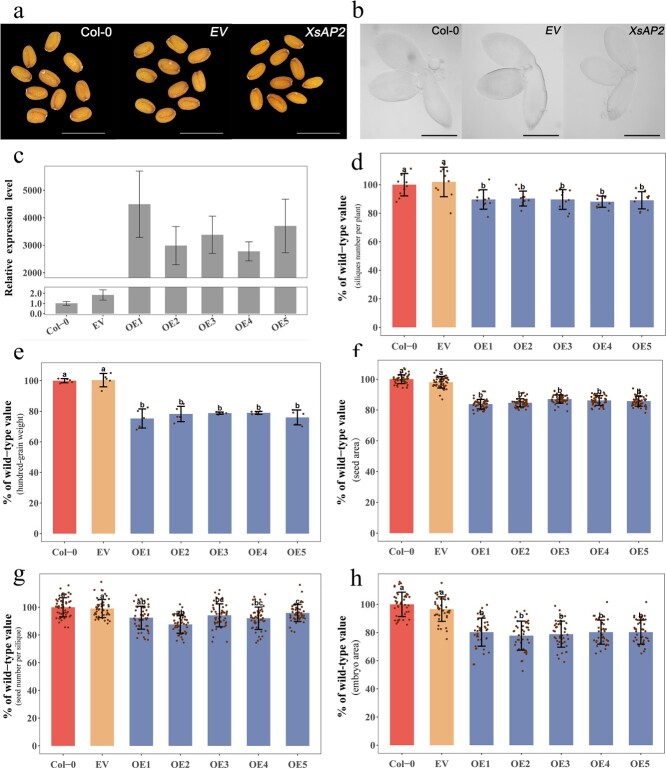
**a** Seeds of Col-0, EV, and *CaMV35S:XsAP2* transgenic *Arabidopsis* (scale bar, 1 mm). **b** Seed embryos of WT, EV, and *CaMV35S:XsAP2* transgenic *Arabidopsis* (scale bar, 300 μm). **c** Relative expression levels of the *XsAP2* gene in WT, EV, and five *XsAP2* transgenic lines. **d** Silique number per plant in WT, EV, and five transgenic lines (*n* = 10). **e** Hundred-grain weight of seeds in WT, EV, and five transgenic lines (*n* = 5). **f** Orthographic projected area of seeds in WT, EV, and five transgenic lines (*n* = 50). **g** Single-fruit seed number in WT, EV, and five transgenic lines (*n* = 50). **h** Orthographic projected area of seed embryos in WT, EV, and five transgenic lines (*n* = 40). Letters above each bar in the graph indicate significant differences (*P* < .001, Tukey’s HSD) compared with WT. Col-0, wild-type *Arabidopsis* with a genetic background of Col-0; EV, *Arabidopsis* transformed by an empty gene vector; OE1, OE2, OE3, OE4, OE5, five transgenic *Arabidopsis* lines overexpressing the *XsAP2* gene.

To explore the influence of *XsAP2* on seed size and yield, we engineered a *CaMV35S:XsAP2* overexpression vector and incorporated it into *A. thaliana* with a Col-0 genetic background. After multigenerational screening, five pure transgenic lines (OE1–OE5) were derived. Prior to the phenotypic observation of these lines, we examined *XsAP2* gene expression levels. Compared with the WT (Col-0) and empty vector (EV) plants, the transgenic lines exhibited a several thousand-fold increase in *XsAP2* expression, affirming successful overexpression of the gene (Fig. 8c). Examination of *Arabidopsis* seeds revealed variations in seed size across the plant. To evaluate the effect of the *XsAP2* gene on seeds more precisely, we observed siliques from the same position on the main branch. Compared with WT and EV, the transgenic lines produced smaller and lighter seeds (Fig. 8a). Specifically, seed area was reduced by 12.9–16.2% and weight per 100 seeds was lightened by 21.2–24.7% (Fig. 8e and f). In mature *Arabidopsis* seeds, the embryo occupies the majority of the volume. We further inquired about the impact of the *XsAP2* gene on seed embryos. The results showed that the area of the seed embryo in the five transgenic *Arabidopsis* lines was reduced by 19.7–22.1% compared with WT and EV (Fig. 8b and h). Despite the variation in the reduction ratios of seed area, embryo area, and seed weight in transgenic *Arabidopsis*, considering that seed area and embryo area do not take the thickness into account, we believe these factors do not conflict. Rather, they collectively suggest that overexpression of the *XsAP2* gene reduces the size of *Arabidopsis* seeds.

In general, seed size and quantity maintain a balance, whereby a reduction in seed size could potentially lead to a surge in seed quantity. Further investigation into the influence of the *XsAP2* gene on the number of seeds per silique and total siliques per plant in *Arabidopsis* was conducted. Unexpectedly, compared with WT and EV, the five transgenic lines demonstrated a reduction of 4.1–12.1% in seeds per silique, and a 9.7–11.9% decrease in the total number of siliques per plant (Fig. 8d and g). These findings suggest that overexpression of the *XsAP2* gene not only results in smaller seeds but also does not facilitate a compensatory augmentation in seed quantity.

## Discussion

### Impact of different models on genome-wide association study analysis

Since the commencement of the first GWAS [27], it has developed into an effective tool for the study of the genetic basis of complex traits and has been extensively used in the identification of economically valuable agronomic traits [28, 29]. GWAS analyses frequently yield false negatives and false positives, attributable to interspecies variability and the complexity of genetic trait relationships [30]. Consequently, selecting an appropriate association model is crucial [31]. We employed four types of association models to conduct the GWAS analysis in this study and found significant variation in the results generated by these models. The multi-locus models (BLINK and FarmCPU) identified only a few significant loci, while the single-locus models (GLM and MLM) identified numerous significant loci (Supplementary Data Table S4). Regrettably, the results from GLM and MLM mostly presented false associations, with most identified candidate genes unrelated to the studied traits. This could be attributed to overfitting of the model, with similar outcomes present in other studies as well [32]. Despite the few loci identified, the FarmCPU and BLINK models had success in identifying the dependable candidate gene *XsAP2* (Figs 4 and 5, Supplementary Data Fig. S2). These results suggest that the FarmCPU and BLINK models are more suitable for GWAS studies on seed size in yellow horn. This finding may also have implications for the study of other traits in yellow horn through GWAS.

### Polymorphisms at the Chr10_24013014 and Chr_24012613 loci affect *XsAP2* gene expression

In this study, based on the resequencing of 222 yellow horn germplasms, we aimed to dissect the molecular mechanisms underlying yellow horn seed yield. In the GWAS analysis of three key factors affecting yellow horn seed yield (HWG, SFSM, and SFSN), except for SFSN, which did not show significant associations with loci, both HWG and SFSM were associated with several significant loci. The Chr10_24013014 and Chr10_24012613 loci on chromosome 10 drew our attention due to their repeated associations (Supplementary Data Table S4). Considering the LD genetic distance, we identified candidate gene *XsAP2* downstream of these two loci. The Chr10_24013014 and Chr10_24012613 loci are in the *XsAP2* gene promoter region, leading us to hypothesize that mutations at these loci may affect the expression activity of the *XsAP2* gene. To test this hypothesis, we selected nine large seed germplasms and seven small seed germplasms and determined their genotypes at the Chr10_24013014 and Chr10_24012613 loci through Sanger sequencing. The results showed that the genotypes of the large-seed germplasm at these loci were CC and CC, while the small-seed germplasm had genotypes −C and GC at these loci (Supplementary Data Fig. S4). These results were consistent with the GWAS analysis. The tissue expression profile of *XsAP2* indicated a high expression level during seed development (DAP40). Therefore, we quantified *XsAP2* gene expression at this stage using RT–qPCR . The results revealed that the expression of the *XsAP2* gene was significantly lower in the large-seed germplasm compared with the small-seed germplasm (Fig. 6f). We further conducted a correlation analysis between the relative expression of the *XsAP2* gene and phenotypic data, demonstrating a strong negative correlation between the relative expression of the *XsAP2* gene and seed weight (HWG, *R* = −0.72) and single-fruit seed yield (SFSM, *R* = −0.71) (Fig. 6g). These findings to some extent confirm that the Chr10_24013014 and Chr10_24012613 loci can influence the expression of the *XsAP2* gene, subsequently affecting yellow horn seed size and single-fruit yield.

### The *XsAP2* gene functions to regulate seed size and yield

The *AP2* transcription factors serve multifarious roles in plant growth and development [33, 34]. This study identifies an *AP2* gene impacting seed size and single-fruit yield through GWAS analysis in yellow horn and confirms its biological role. Tissue expression analysis revealed that the *XsAP2* gene was expressed in various tissues of yellow horn, with higher expression in flowers and seeds, particularly during the rapid seed growth period (Fig. 7a). This suggests that the *XsAP2* gene is involved in flower and seed development. To further explore the biological function of *XsAP2*, we overexpressed the *XsAP2* gene in WT *Arabidopsis*. Compared with WT and EV, the *XsAP2* transgenic lines showed different reductions in seed area, seed HWG, and embryo area (Figure 8e, f, and h). The results indicate that *XsAP2* has a negative regulatory effect on the size of the seed. This function exhibits similarity to the *Arabidopsis AP2* gene [12, 13, 35]. Additionally, a slight reduction in the number of siliques per plant and the number of seeds per silique occurred in transgenic *Arabidopsis* compared with WT and EV (Fig. 8d and g). The number of fruits is correlated with the number of flowers. The function of the *Arabidopsis AP2* gene is best known as that of a class A gene in the ABCED model of flower development. It antagonizes class C genes and controls the morphogenesis of floral organs [36, 37]. In addition, the *XsAP2* gene is a homolog of the *Arabidopsis AP2* gene (Supplementary Data Fig. S5). The above results indicated that overexpression of the *XsAP2* gene negatively regulated seed size and yield in *A. thaliana*, proving that the *XsAP2* gene has the function of regulating seed size and yield. In addition, both HWG and SFSM of yellow horn had a strong correlation with the relative expression of the *XsAP2* gene (Fig. 6g). Therefore, we hypothesized that the *XsAP2* gene also had some negative regulatory effects on seed size and single-fruit yield of yellow horn.

## Conclusions

Our study has elucidated that mutations at the Chr10_24013014 and Chr10_24012613 loci have the capacity to influence the expression activity of the *XsAP2* gene. Furthermore, the *XsAP2* gene exerts a certain degree of negative regulation on yellow horn seed size and single-fruit yield. These findings unveil the genetic regulatory mechanisms governing yellow horn seed size and yield, laying the groundwork for the development of molecular markers for early identification of high-yielding yellow horn plants and providing valuable insights for molecular breeding aimed at achieving higher yellow horn yields.

## Materials and methods

### Phenotype data collection and quality control

Following seed maturation, a total of 143 and 222 sample trees were surveyed at the yellow horn Germplasm Resource Nursery, located in Tongliao City within the Inner Mongolia Autonomous Region, China, during the years 2020 and 2022, respectively. (Special note: the 222 sample trees surveyed in 2022 fully covered the 143 sample trees surveyed in 2020.) Six fruits were harvested from each sample tree, and each fruit served as a biological replicate. The total mass of single-fruit seeds (referred to as SFSM) was measured, and the number of seeds per single fruit (referred to as SFSN) was recorded. Subsequently, the hundred-grain weight (referred to as HWG) of the seeds was computed. The coefficient of variation among the six biological replicates for each sample tree was calculated, with the exclusion of outliers to maintain a coefficient of variation <0.1. The mean of the phenotypic data of the six fruits was then calculated as the final phenotypic data for this sample tree. Following this, the phenotypic data for SFSM, SFSN, and HWG were subjected to a Kolmogorov–Smirnov test to assess normality. Any outlier samples were removed to ensure the reliability of phenotypic data for subsequent GWAS analyses. Furthermore, a linear regression analysis was conducted to investigate the consistency of the same phenotype between the two years.

### Plant materials and genome resequencing

Tissue samples were collected from young and healthy leaves and snap-frozen in liquid nitrogen, and the samples were ground into fine powder using a ball mill. Genomic DNA of yellow horn leaves was extracted using the CTAB method and assessed for quality. Genomic DNA sequences were fragmented using ultrasound and sequentially subjected to DNA end repair, poly-A addition at the 3′ end, phosphorylation at the 5′ end, ligation of junctions, PCR amplification, and magnetic bead purification to construct a qualified sequencing library. Subsequently, double-end sequencing (PE150) was performed using the Illumina platform (HiSeq 4000). Finally, the data were quality-checked using fastp (version 0.23.4) software [38].

### SNP calling and quality control

Four yellow horn reference genomes have been published: ZS4 [39], JGXP [40], WF18v1 [41], and WF18v2 [42]. Given the closer relationship between the sequenced samples and ZS4 yellow horn, we selected the ZS4 genome as our reference genome. This genome has 15 chromosomal pseudomolecules, in which 97.04% of the sequences are anchored, with a scaffold N50 size of 32.17 Mb, a contig N50 size of 1.04 Mb, and complete BUSCO value of 98.7%, and 24 672 protein-coding genes have been successfully annotated. These evaluation results confirm that the assembly and annotation of this genome exhibit a high quality level, rendering it suitable for use as a reference genome. Subsequently, Trimmomatic software (version 0.39) [43] was employed to eliminate adapters from the reads and filter out low-quality sequences. BWA software (version 0.7.17) [44] was utilized for mapping the reads to the reference genome. Picard software (version 3.0.0) was employed to sort the reads and eliminate PCR amplification-induced duplicates. GATK software (version 4.4.0.0) [45] was utilized for SNP and indel variant locus calling. Plink software (version 2) [46] was used to calculate the minimum allele frequency (MAF = 0.05), SNP genotyping deletion rate (geno = 0.1), and Hardy–Weinberg equilibrium filtering (hwe = 1e−06).

### Genome-wide association analysis and identification of candidate genes

GWAS was conducted using GAPIT3 [47] to explore the associations between three phenotypes (SFSM, SFSN, and HWG) and a total of 2 164 863 high-quality variant loci in 2020 and 2022, respectively. Bayesian-information and Linkage-disequilibrium Iteratively Nested Keyway (BLINK) [48], Fixed and random model Circulating Probability Unification (FarmCPU) [49], Mixed Linear Model (MLM) [50], and General Linear Model (GLM) [51] were the models employed for the analysis. The BLINK model and the FarmCPU model are multi-bit point models, which outperform the single-point models MLM and GLM in terms of both statistical power and computational efficiency [47]. Additionally, to mitigate the impact of population stratification-induced false positives, PCA was conducted on the genotype matrix, and the first three principal components were included as covariates in the GWAS analysis. Meanwhile, kinship matrices constructed using the VanRaden method [52] were incorporated into the GWAS analysis to mitigate spurious associations resulting from sample kinship. The Bonferroni method was applied as a correction for multiple hypothesis testing. The association between SNP loci and phenotypes was evaluated using −log10(0.05/*k*) as the significance threshold, with *k* representing the total number of variant loci. Manhattan and QQ plots were created by using the qqman package for R software (version 4.2.0) to visualize GWAS results. In addition, the level of LD was determined by calculating the LD coefficient using PopLDdecay software (version 3.42) [53], and the LD decay distance was defined as the distance where the LD coefficient *r*^2^ reached half of its maximum value.

To identify candidate genes with high confidence, the significant SNP loci identified in the GWAS analysis were further filtered, retaining those that were called multiple times. Subsequently, a reference LD decay distance was utilized to identify candidate genes located within 20.75 kb upstream and downstream of the significant SNP loci. Finally, gene functions were queried using the NCBI database to determine the ultimate candidate genes.

### DNA sequencing and gene expression analysis

To validate the accuracy of the GWAS analysis, specific primers were designed to clone the promoter region of the *XsAP2* gene in nine germplasm samples with small HWG and seven samples with large HWG. Primer sequences can be found in Supplementary Data Table S5. Subsequently, these germplasms were sequenced using the Sanger method to determine the genotypes of the Chr_24012613 and Chr_24013014 loci. In addition, to verify the effect of the genotypes of these two loci on the expression of the *XsAP2* gene, the expression of the *XsAP2* gene in these germplasms at the seed (DAP40) stage was determined by RT–qPCR.

### Subcellular localization

The coding sequence of the *XsAP2* gene without a stop codon was amplified using the T vector containing the target gene as a template and inserted into the *pCAMBIA35S-eYFP* expression vector. In addition, the *H_2_B* histone gene served as a nuclear marker. *Agrobacterium tumefaciens* (GV3101, Sangon Biotech, China) was separately transformed with three plasmids: *35S:XsAP2-eYFP*, *35S:H_2_B-RFP*, and *35S:eYFP*. Subsequently, *N. benthamiana* leaves were instantaneously transformed. Following 12 h of dark treatment and 24 h of normal treatment, the yellow (eYFP) and red (mCherry) fluorescent signals were visualized using a laser confocal microscope (Zeiss LSM880 Airyscan FAST+ NLO).

### Phylogenetic tree construction

Protein sequences of the *Arabidopsis AP2/ERF* gene family were downloaded from The Arabidopsis Information Resource website (https://www.arabidopsis.org/). The protein sequences of the *Arabidopsis AP2/ERF* gene family and *EVM0013598* gene were aligned using Clustal X software for multiple sequence alignment. Subsequently, MEGA11 software [54] was used to construct the phylogenetic tree employing the neighbor-joining method. The specific parameters used were: 1000 bootstrap, JTT + G model, and partial deletion (50%).

### Generation of transgenic *A. thaliana*

The full coding sequence of the *XsAP2* gene was amplified using yellow horn flower cDNA as a template and inserted into the *pGEM-T* vector (A1360, Promega, USA). Sequencing was performed using universal primers (SP6/T7) to ensure sequence accuracy. The correct coding sequence was cleaved from the T vector and ligated between the BglII and MluI restriction sites of the *pCAMBIA35S-eYFP* vector (Supplementary Data Fig. S6). Subsequent sequencing was conducted to confirm the accuracy of the sequence. The *CaMV35S:XsAP2* plasmid was introduced into WT *Arabidopsis* (Col-0) by *A. tumefaciens* (GV3101, Sangon Biotech, China) using the inflorescence infestation method. The putative *F*_1_ generation transformant seedlings were identified using glufosinate ammonium (100 mg/l). Leaf tissue genomes of *F*_1_-generation transformants were extracted, amplified by PCR using universal primers specific to the *pCAMBIA35S-eYPF* vector, followed by sequencing to verify sequence integrity and positive identification. Seeds from each *F*_1_-generation transformed line were individually collected and transplanted. The *F*_2_-generation transformants were further screened with glufosinate ammonium salt (100 mg/l), and only lines exhibiting a 3:1 pattern of trait segregation was chosen. Subsequently, *F*_2_-generation transformants were cultivated under appropriate growth conditions, and *F*_3_-generation seeds were collected on a plant-by-plant basis. From the seeds harvested from each *Arabidopsis* plant, a subset of seeds (~100 seeds) was randomly selected for replanting. Seedlings were once again screened using glufosinate ammonium salt (100 mg/l). The identity numbers of *Arabidopsis* plants with fully surviving seedlings were recorded. These plants are considered homozygous and suitable for subsequent phenotypic observation. Additionally, WT and EV *Arabidopsis* plants were cultured under identical conditions as the control. All vector sequences and primer sequences are detailed in Supplementary Data Table S3.

### Phenotypic observation of transgenic *Arabidopsis* seeds

Five identified *XsAP2* transgenic lines of *A. thaliana* were selected for observation. First, the total number of siliques per plant was counted. Subsequently, the 1st to 10th siliques (counting from the base of the main branch) on the main branch from each *Arabidopsis* plant were selected. The seeds inside each selected silique were photographed using orthographic projections at a consistent scale. The number and orthographic area of seeds were quantified using ImageJ software. The seed weight of 10 siliques from each *Arabidopsis* plant was measured using a high-precision electronic balance with an accuracy of 1/10 000. The weight of 100 seeds was determined by calculation. As controls to evaluate the impact of the *XsAP2* gene, contemporaneous WT *Arabidopsis* plants and EV *Arabidopsis* plants were selected.

### Microscopic observation of *Arabidopsis* embryo

Embryo specimens were prepared following the method established by previous researchers [10, 12]. After specimen preparation, *Arabidopsis* embryos of different lines were photographed at the same magnification using a digital microscope (Motic, M17T-HD-P). The projected area of the embryos was subsequently calculated using ImageJ software.

### RT–qPCR analysis

To examine the tissue-specific expression patterns of the *XsAP2* gene in yellow horn, we collected tissue samples from seeds, roots, stems, leaves, and flowers at various developmental stages. Considering the potential impact of polymorphisms at the Chr10_24012613 and Chr10_24013014 loci, located in the promoter region, on the tissue-specific expression profile of the *XsAP2* gene, we collected tissue samples from small-seed, normal-seed, and large-seed germplasm.

To determine the correlation between phenotypic changes in seeds of transgenic *A. thaliana* and *XsAP2* gene expression levels, seed tissues were collected from five transgenic lines, as well as from WT and EV of *A. thaliana*.

Total RNA was extracted by the CTAB method from all the above collected tissue materials. First-strand cDNA was synthesized using the TransScript-Uni One-Step gDNA Removal and cDNA Synthesis SuperMix kit (AU311, Trans, China). Real-time quantitative PCR analysis was conducted using the TB Green Premix Ex Taq II kit (RR820Q, TaKaRa, Japan) on a LightCycler 480 system (three biological replicates and four technical replicates). The reaction volume was 10 μl and the cDNA concentration was 100 ng/μl. The β-actin genes of *A. thaliana* and yellow horn were used as internal controls. Relative gene expression was calculated using the 2^−∆∆Ct^ method. Gene primer sequences, internal reference primer sequences, and detailed reaction conditions are available in Supplementary Data Table S5.

### Statistical analysis

Differences between groups were analyzed using one-way ANOVA, followed by *post hoc* multiple comparisons using Tukey’s method. The correlation between groups was analyzed using Pearson’s correlation coefficient. All analyses were conducted using the R environment (version 4.2.0).

## Supplementary Material

Web_Material_uhad243

## Data Availability

The data underlying this article are available in the NCBI database at https://www.ncbi.nlm.nih.gov/sra, and can be accessed with PRJNA1031336. The detailed run code and parameters for the GWAS analysis are available on GitHub (https://github.com/ziquanzhao/Python_GX/tree/master/Python_apply/EasyGWAS).
