# Supplementary material for: Genome-wide association analysis identifies a candidate gene controlling seed size and yield in Xanthoceras sorbifolium Bunge
Source: Hortic Res. 2023 Nov 22;11(1):uhad243. doi: 10.1093/hr/uhad243 (PMC10788774; doi:10.1093/hr/uhad243)
Supplement: Web_Material_uhad243 [file web_material_uhad243.zip › Figure S4.pdf]

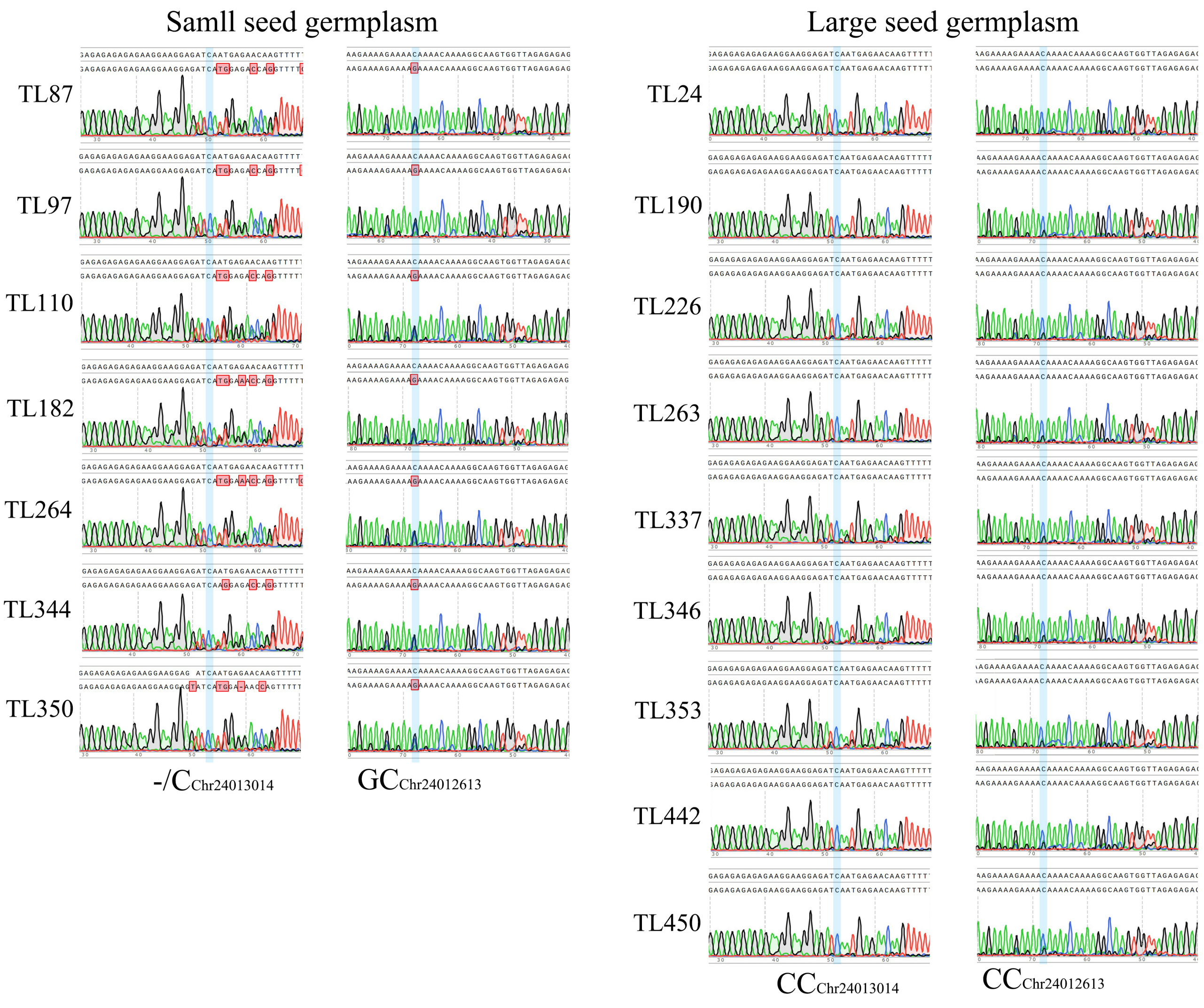
Samll seed germplasm
Large seed germplasm
TL87
TL97
TL110
TL182
TL264
TL344
TL350
TL24
TL190
TL226
TL263
TL337
TL346
TL353
TL442
TL450
-/C Chr24013014
GC Chr24012613
CC Chr24013014
CC Chr24012613
