# Supplementary figures and images for: Genome-wide association analysis identifies a candidate gene controlling seed size and yield in Xanthoceras sorbifolium Bunge
Source: Hortic Res. 2023 Nov 22;11(1):uhad243. doi: 10.1093/hr/uhad243 (PMC10788774; doi:10.1093/hr/uhad243)

Color Key  
and Histogram

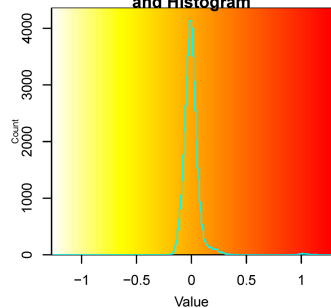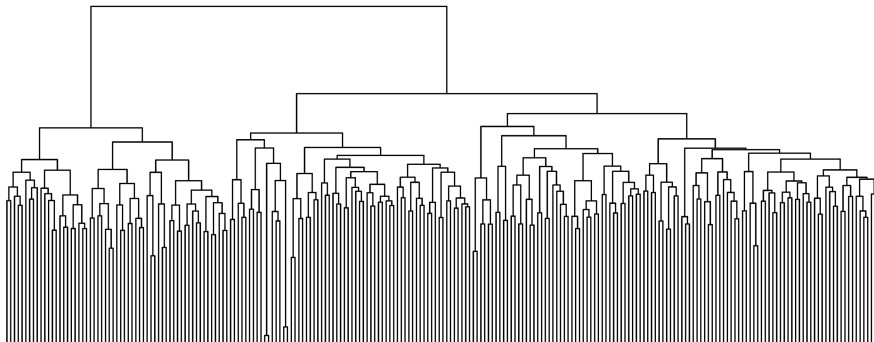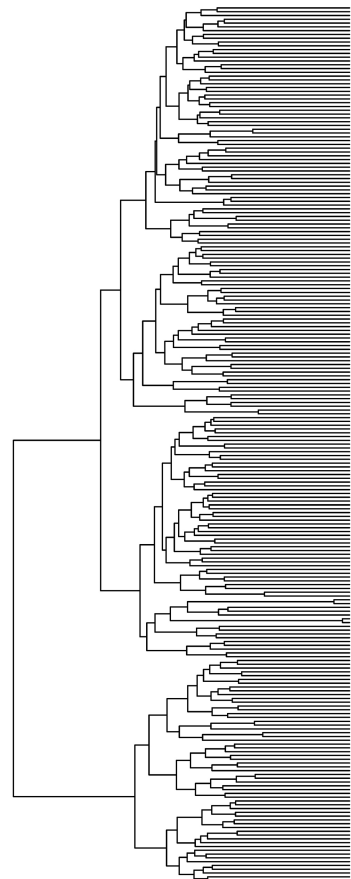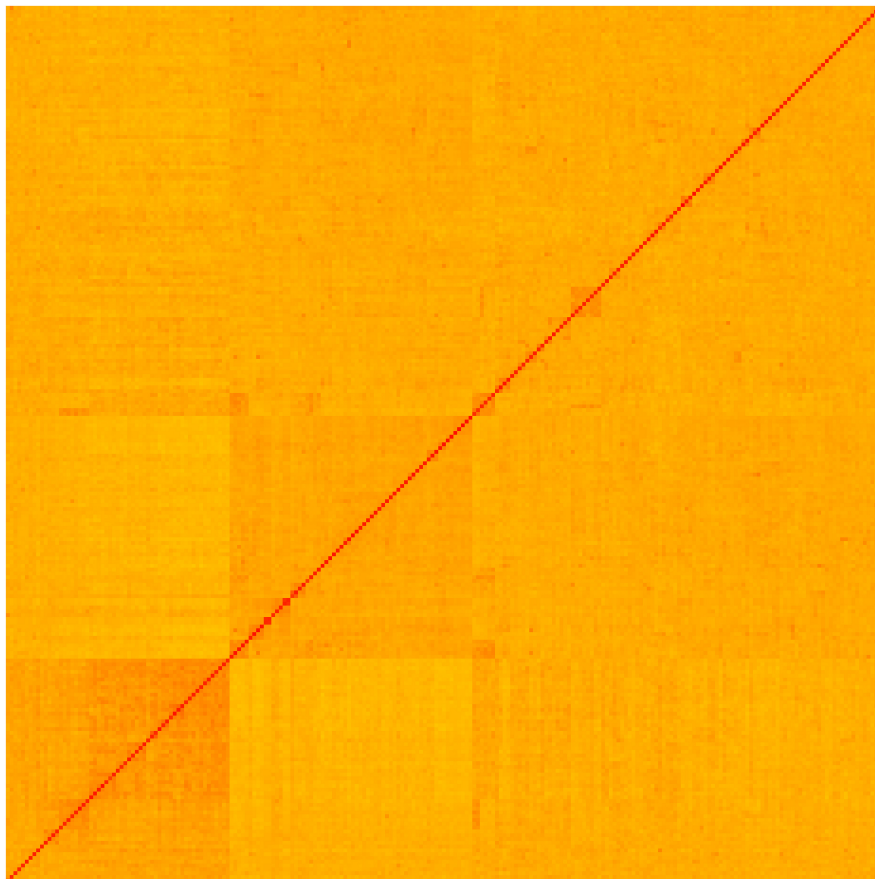

Supplement: Web_Material_uhad243 [file web_material_uhad243.zip › Figure S1.pdf]

SFSN2022

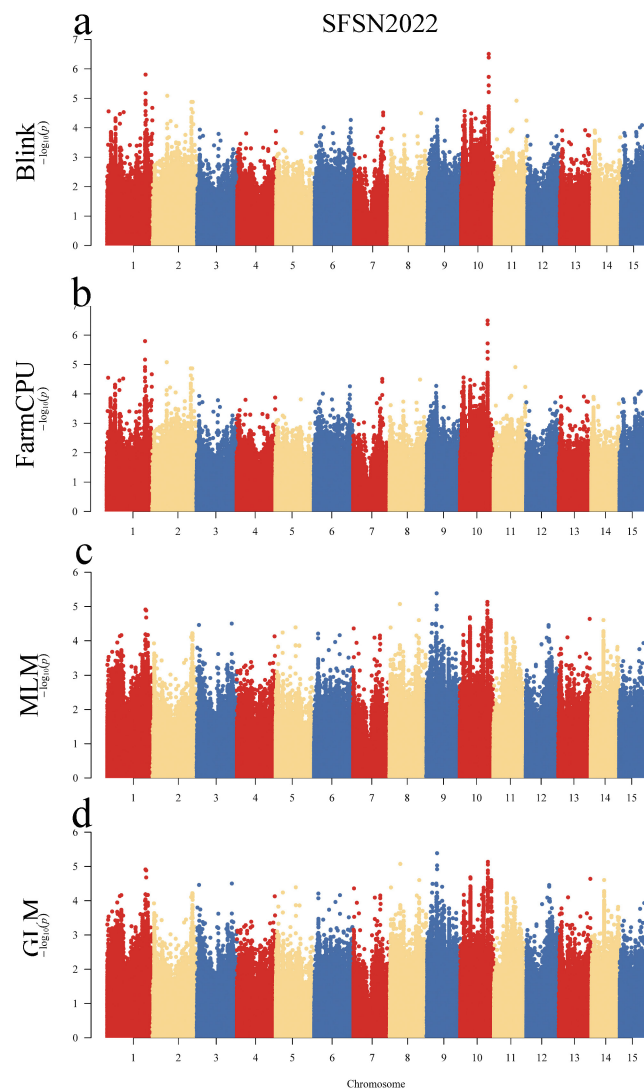

SFSN2020

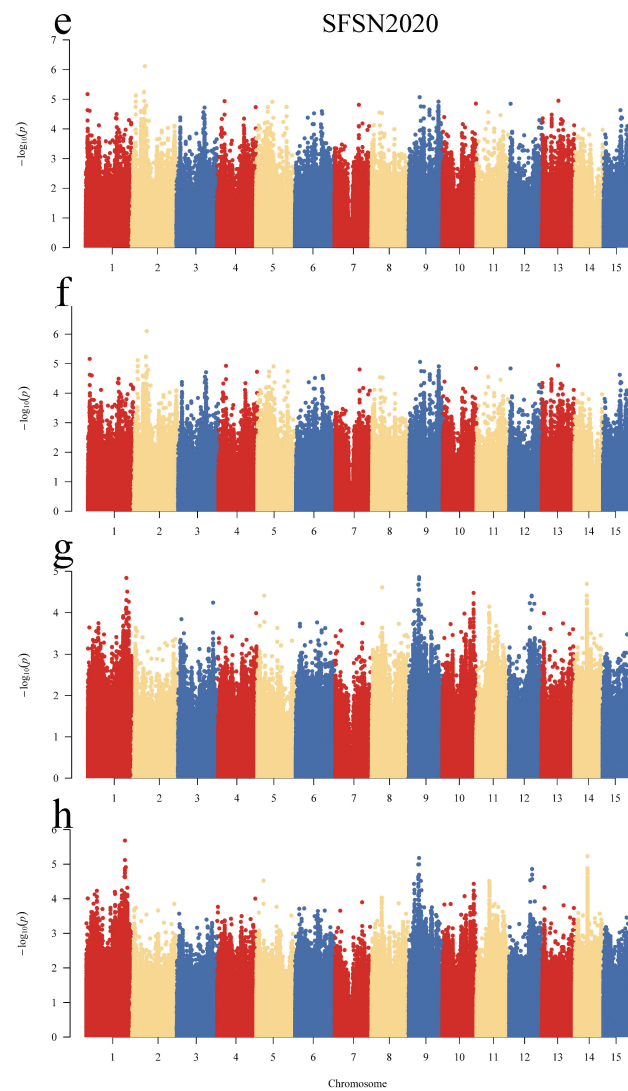

Supplement: Web_Material_uhad243 [file web_material_uhad243.zip › Figure S2.pdf]

## Blink

FarmCPU

MLM

GLM

HWG2022

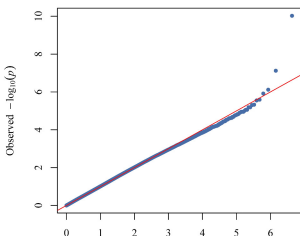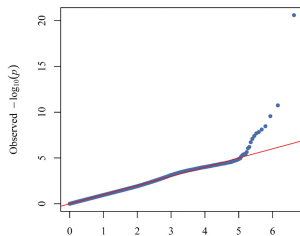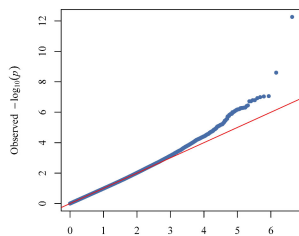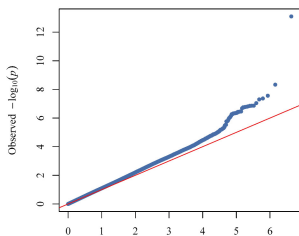

HWG2020

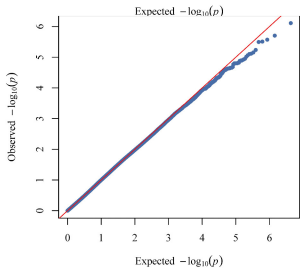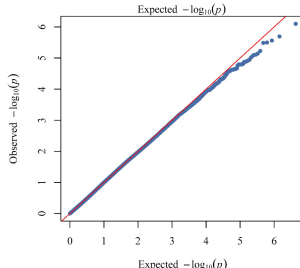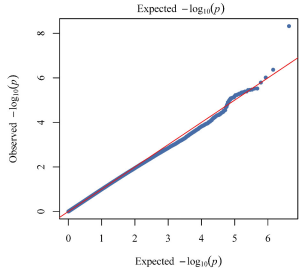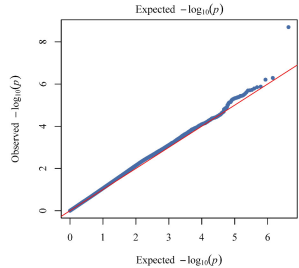

SFSM2022

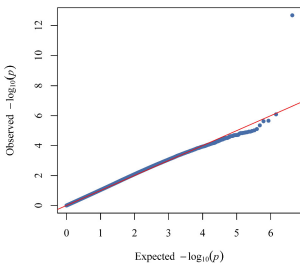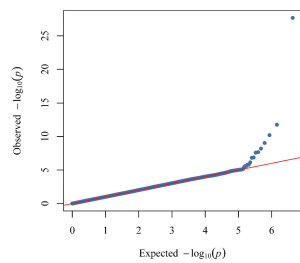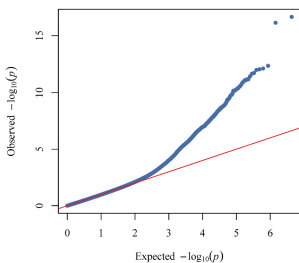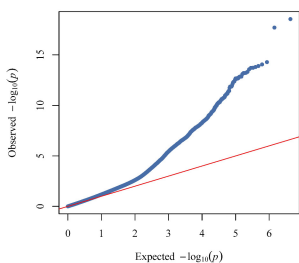

SFSM2020

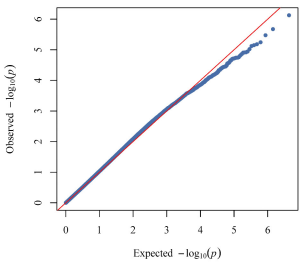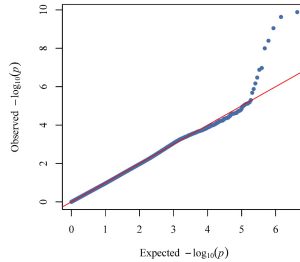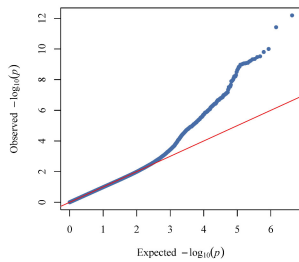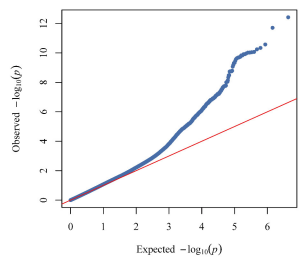

SFSN2022

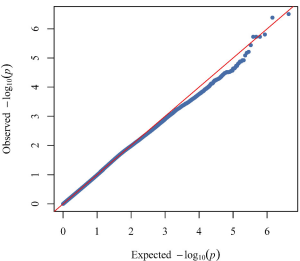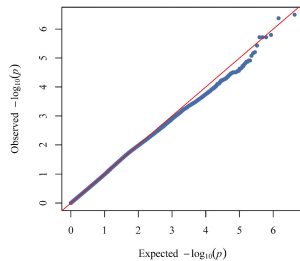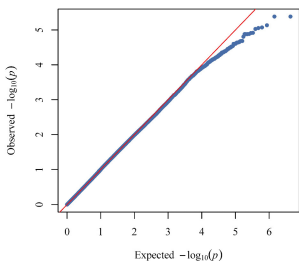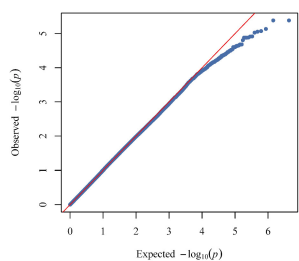

SFSN2020

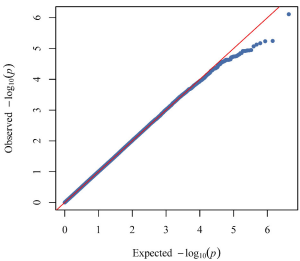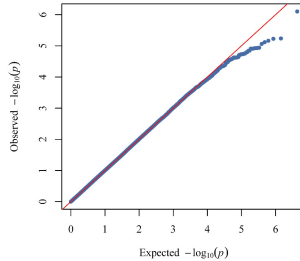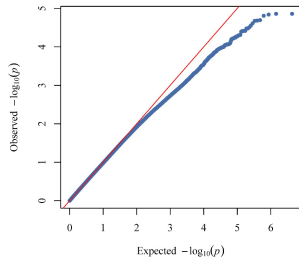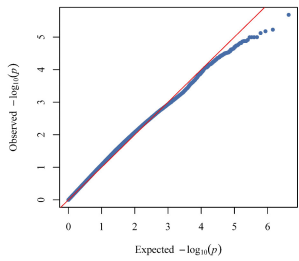

Supplement: Web_Material_uhad243 [file web_material_uhad243.zip › Figure S3.pdf]

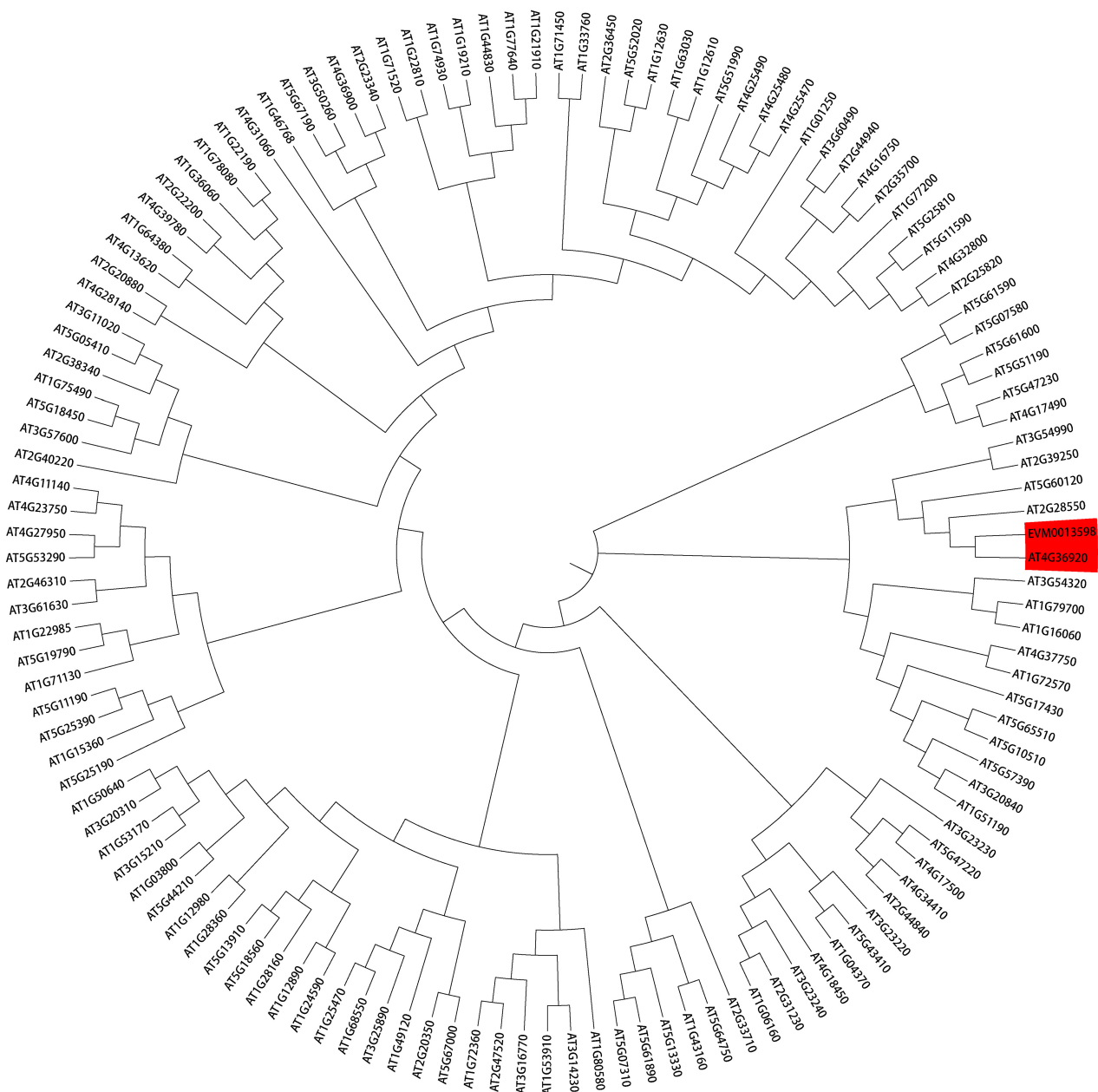

Supplement: Web_Material_uhad243 [file web_material_uhad243.zip › Figure S5.pdf]

Empty vector

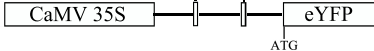

Expression vector

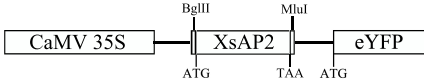

Supplement: Web_Material_uhad243 [file web_material_uhad243.zip › Figure S6.pdf]
